# Rapidly Progressive Orbital Apex Syndrome Due to Scedosporium apiospermum Following Endoscopic Sinus Surgery

**DOI:** 10.7759/cureus.18541

**Published:** 2021-10-06

**Authors:** Steven R Engebretsen, Luxman Srikantha, Samba Siva Bathula

**Affiliations:** 1 Otolaryngology - Head and Neck Surgery, Detroit Medical Center, Michigan State University, Detroit, USA; 2 Otolaryngology, Detroit Medical Center, Michigan State University, Detroit, USA

**Keywords:** orbital cellulitis, paranasal sinuses, fungal disease, sinus infections, sudden vision loss

## Abstract

*Scedosporium apiospermum* is a ubiquitous, highly resistant opportunistic fungus found in sewage and polluted waters and may infect the paranasal sinuses. Orbital Apex Syndrome may occur following trauma, surgery, or infection. An 80-year-old male with diabetes mellitus and mild dementia underwent uncomplicated, bilateral functional endoscopic sinus surgery for chronic sinusitis with polyposis. Initial pathology was reported as non-invasive bacterial and fungal species. On postoperative day 4, he had sudden right vision loss and abducens nerve palsy. Imaging noted violation of the lamina papyracea and inflammation of the optic nerve without compression. Medical therapy was begun and the patient developed sudden vision loss of the left eye. The patient then underwent emergent surgical decompression of both optic nerves. A final culture from the original surgery of *S. apiospermum* was made on postoperative day 10. Aggressive medical therapy was continued and the patient ultimately expired from complications of medical therapy and other underlying conditions. Trauma to the delicate bony walls of the orbit during sinus surgery in an immunocompromised patient who is unknowingly colonized with *S. apiospermum* can lead to the rapid spread of this highly neurotoxic organism.

## Introduction

*Scedosporium* *apiospermum* is a ubiquitous, highly resistant, and opportunistic fungus that may infect the paranasal sinuses and nervous system [1-3]. Although rarely reported, it is increasingly being noted in certain parts of the world [4] and found worldwide [5]. *Scedosporium* has been noted as one of the underrated opportunistic infections in a recent review [5]. Orbital apex syndrome (OAS) is characterized as the constellation of symptoms including unilateral visual loss and ophthalmoplegia of multiple cranial nerves. This is a reported case of *S.* *apiospermum* induced OAS following functional endoscopic sinus surgery (FESS) with devastating consequences.

## Case presentation

**Figure 1:**
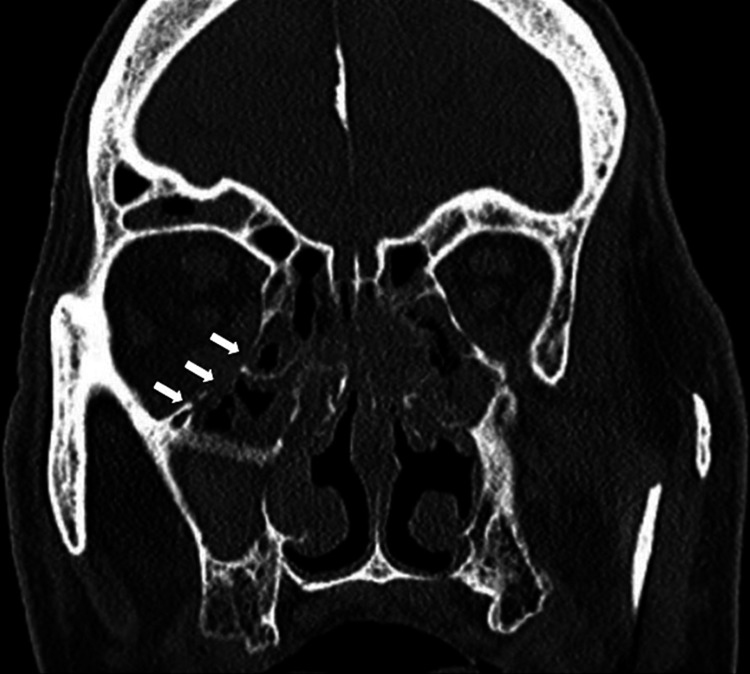
Violation of the lamina papyracea creating communication between the sinus and orbital apex (arrows).

An 80-year-old male with diabetes mellitus, mild diabetic retinopathy, and mild dementia underwent reported uncomplicated, bilateral FESS for chronic sinusitis with polyposis at an outside hospital. He was provided an outpatient prescription for amoxicillin-clavulanic acid upon outpatient surgical discharge. Initial pathology specimen within the immediate postoperative period from the outside hospital was reported as non-invasive *Aspergillus*. He was seen by his original surgeon at the outside facility on postoperative day 3 without visual complaints or unexpected facial pain. On postoperative day 4, the patient reported sudden right-sided vision loss and presented to the walk-in ophthalmology clinic at the primary author’s tertiary care facility. An ophthalmology exam found no perception of light on the right and an abducens nerve palsy. Pupils were equal and reactive to light bilaterally with no pallor of the optic disc and normal ocular pressures. He was sent immediately to the emergency department for imaging, whereupon the otolaryngology team evaluated the patient. Intranasal exam by otolaryngology was only notable for moderate edema of the nasal mucosa without necrosis or pallor. There was no decreased sensation of the palate or midface. Computed tomography was performed and notable for violation of the lamina papyracea (Figure 1). Preoperative imaging from the outside facility was not available for comparison. Magnetic resonance imaging suggested acute ischemic findings of the right optic nerve with diffusion restriction at the proximal optic nerve without evidence of thrombosis or hemorrhage. Subsequent imaging after admission noted an abnormal T2 signal without canalicular segment enhancement. There was, however, some adjacent enhancement extending to involve the optic and nerve sheaths in the orbital apex and superior orbital fissure. The enhancement of the right-sided nerve sheaths was thought to be contributing both to the vision loss and the abducens nerve palsy that was defining the OAS. In addition to those mentioned, the neurosurgery, neurology, and internal medicine teams evaluated the patient. It should be noted that repeated images were obtained at several time points due to poor images from motion artifact that did delay care. The clinical and radiologic findings were determined by the treating teams to be most suggestive of the surgical trauma from the FESS at the outside facility and not of an infectious origin. A non-surgical approach was initially pursued. The patient was provided broad-spectrum antibiotics and high-dose steroids to decrease inflammation with close neurologic monitoring. Re-evaluation by otolaryngology on flexible nasal endoscopy did not show any change in findings of the nasal mucosa.

**Figure 2:**
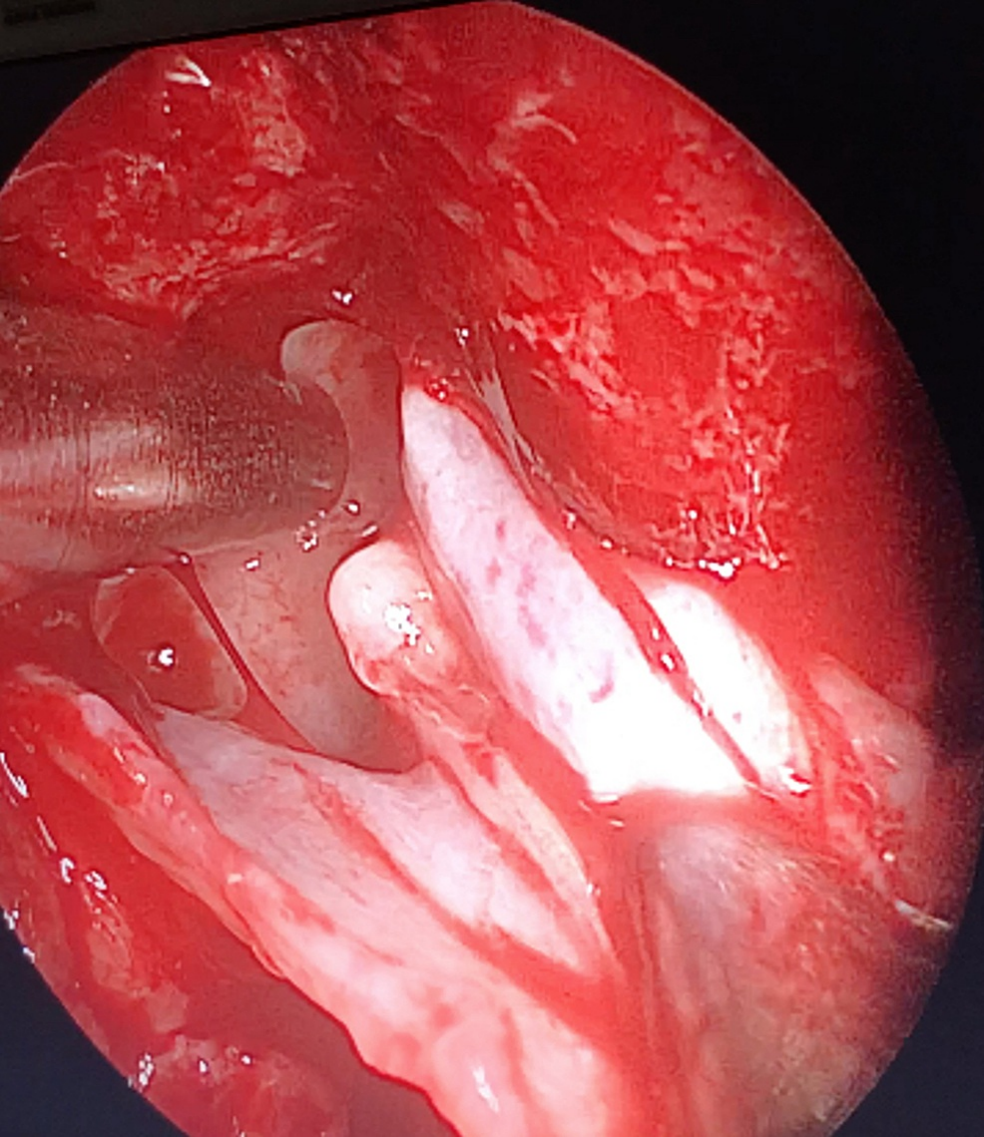
Mucosal stripping intraoperatively.

**Figure 3:**
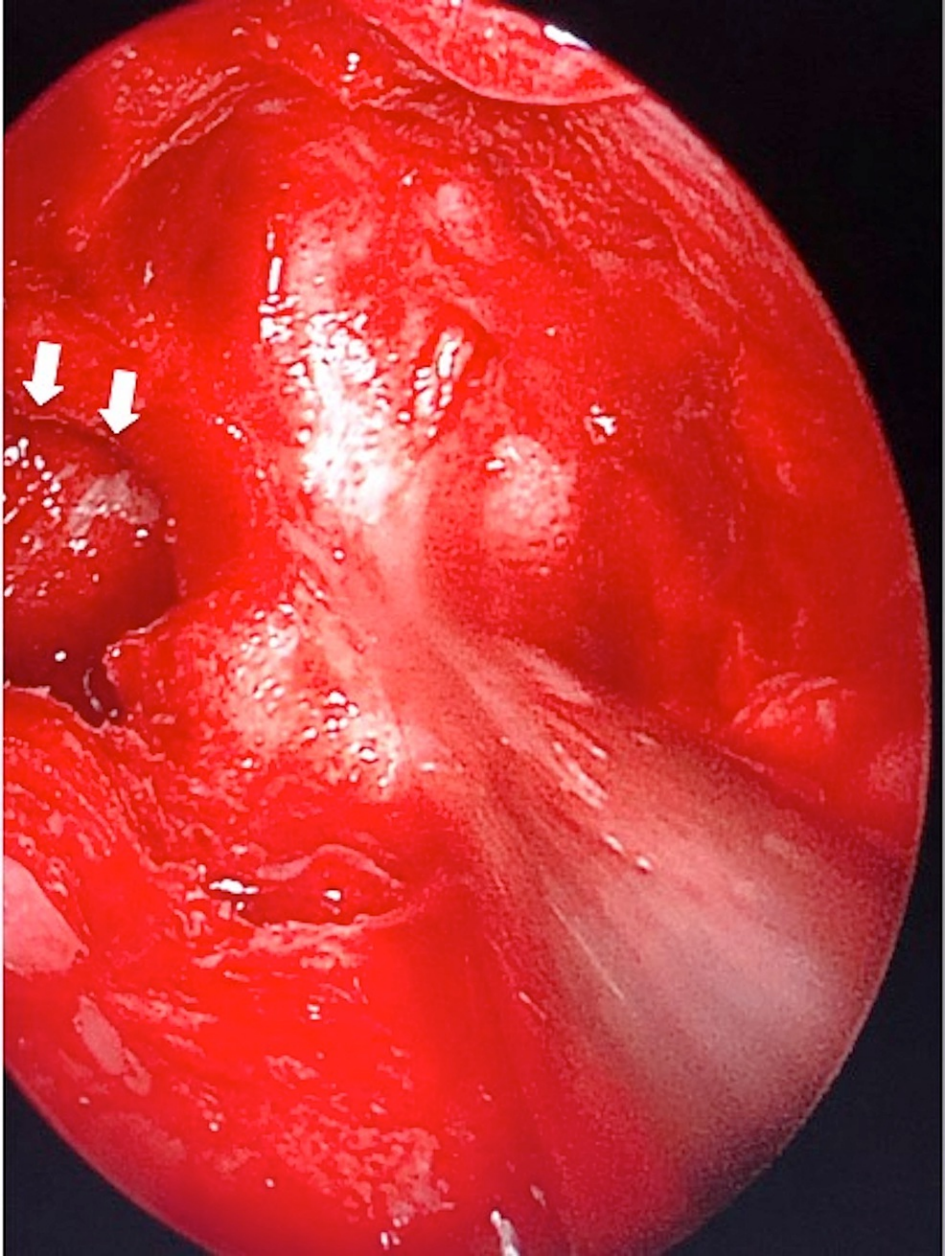
Intraoperative findings of violated lamina papyracea likely pathway of infection to orbital apex (arrows).

On postoperative day 9 from the original FESS, the patient developed sudden vision loss of the left eye. An ophthalmology exam noted no light perception bilaterally, worsening ophthalmoplegia on the right with decreased supraduction, adduction, and infraduction in addition to the complete abducens palsy. These findings suggested now the involvement of the oculomotor and trochlear nerves. The posterior ocular exam now showed mild optic nerve pallor on the right with good color on the left and no evidence of edema. Repeat computed tomography and magnetic resonance imaging noted no new bony destruction; however, there was persistent inflammation of the right optic canal. An emergent multidisciplinary team meeting was held between all consulting specialties and infectious disease was also consulted as the initial diagnosis of traumatic neuropathy was questioned. The patient then underwent emergent FESS with otolaryngology and neurosurgery with decompression of both optic nerves, sphenoidotomy, septectomy, and mucosal stripping (Figure 2). The lamina papyracea and bone adjacent to the root of the right orbital apex were dehiscent as had been suggested on imaging (Figure 3). Additionally, there was a large amount of absorbable packing in the posterior ethmoid cavity on the right.

A final culture from the initial FESS at the outside hospital was re-evaluated by the infectious disease team, which revised the diagnosis of *Aspergillus *to that of *S.* *apiospermum*. This pathologic diagnosis was made on postoperative day 10 (postoperative day 1 from emergent FESS). The patient was started on high-dose voriconazole and amphotericin B. The patient continued on maximum medical therapy and continued to worsen clinically and radiologically regarding the infection. Unfortunately, after prolonged hospitalization, he suffered a myocardial infarction and subsequent severe gastrointestinal bleeding. He passed away on postoperative day 47 from the initial FESS after being transferred to inpatient hospice services.

## Discussion

*S.* *apiospermum* has been reported to cause extensive infections through minor and unrecognized traumatic defects [1]. This organism has also been reportedly resistant to amphotericin B and many of the azole family [5]. Mortality for disseminated infections has been reported to be as high as 50% [1,6] and is clinically noted to be more likely in immunocompromised individuals [7]. The histopathologic diagnosis of this organism may also be inaccurate in up to 22% of cases.

There are several other reports of *Scedosporium* spp. involving the orbit in the literature spanning the last 40 years and most suffered from complications following the infection [6,8,9]. Loh et al. described the most similar situation to the current case involving progressive OAS in the setting of fungal pansinusitis of *S.* *apiospermum* following extraction of abscessed teeth. That patient was treated with multiple FESS interventions. Similar to the current case, he also continued to deteriorate to bilateral vision loss despite maximum medical and surgical therapies [10]. On the contrary, another reported case noted pathologic findings of *S. apiospermum* in the setting of cacosmia with resolution after balloon sinuplasty and no complications [11]. A small case series of fungal sinusitis reported another species called *S.* *prolificans*. All six of these patients presented with either loss of vision, proptosis, or orbital pain. All except for one patient were able to be managed with aggressive medical therapy and surgical debridement sparing orbital exenteration [9].

It is probable that patients in this rare condition are colonized with *Scedosporium *spp. for an unknown time prior to minor trauma. A small number of cases of traumatic etiology leading to *Scedosporium* spp. infection has been reported in the ophthalmology literature [12,13]. In the current case, the routine FESS likely caused a small, unnoticed violation of the thin bony wall over the right orbital apex allowing for the infectious spread in an otherwise quiescent infection. The initial surgical intervention, as well as the imaging in the current case, was at an outside hospital. Thus, only the medical records and phone communication were available for review. Whether the damage to the region in question occurred via surgical instruments or during over-aggressive packing of the nasal cavity with absorbable agents is not known. It is also possible that the infection was present prior and that bony wall destruction was present prior to surgery; however, this cannot be confirmed as the prior imaging was not available for review. Care was also delayed and confounded by the difficulty of identifying *Scedosporium*, which was only accurate after the patient already developed bilateral blindness. It is important to note that the visual appearance of this infected tissue during surgical decompression was subtle and pale, but no obvious necrosis was noted. The initial management of this patient’s blindness presentation was largely considered to be due to surgical trauma. Rare fungal etiology was not a major concern until the patient developed bilateral blindness and repeat imaging showed worsening inflammation despite high dose steroids. Confidence was also placed in a pathology report that ultimately was inaccurate from the initial surgery. With these important and tragic learning points in mind, otolaryngologists and other specialties should understand that a high index of suspicion should be held for the rapid identification and treatment of rare fungal causes in the postoperative setting, especially in the setting of abnormal symptomatology.

## Conclusions

Trauma to the delicate bony walls of the orbit during sinus surgery in an immunocompromised patient who is unknowingly colonized with *Scedosporium *spp. can lead to the rapid spread of this highly neurotoxic organism. Care and a high index of suspicion should be kept in mind for the rapid identification and treatment of this devastating infection.
